# Self-Assembled Nanoparticles of Licorice Extract Enhance Skin Penetration and Regulate Barrier Proteins via a Dual-Channel Pathway

**DOI:** 10.3390/pharmaceutics18060661

**Published:** 2026-05-27

**Authors:** Wenjie Ning, Lingyu Hang, Yuye Xue, Wenting Zha, Run Wang, Kailin Xue, Jiantao Ning, Jiankang Zhao, Liqiang Wang, Hailong Yuan

**Affiliations:** 1 Department of Pharmacy, Air Force Medical Center, People’s Liberation Army of China, Air Force Medical University, Beijing 100142, China; 18850146604@163.com (W.N.); 17610046218@163.com (L.H.); xueyuye0628@126.com (Y.X.); zhawenting2023@163.com (W.Z.); wangrun0824@163.com (R.W.); 13960169603@163.com (K.X.); njt15684198295@163.com (J.N.); 2 School of Medicine, Huaqiao University, Quanzhou 362021, China; mpp5358@163.com

**Keywords:** licorice, self-assembled, transdermal delivery, nano-formulation, atopic dermatitis, barrier proteins

## Abstract

**Objective:** Self-assembled nanoparticles (SANs) naturally occurring in Traditional Chinese Medicine (TCM) decoctions are promising drug carriers due to their biocompatibility, but uncontrolled assembly often leads to poor stability, limiting transdermal permeability and industrial application. This study aimed to fabricate stable and uniform SANs from licorice by precisely regulating the controlled nanoprecipitation of its water- and alcohol-extracted components. The transdermal delivery efficiency and therapeutic efficacy of the SANs in the treatment of atopic dermatitis (AD) were evaluated. **Methods:** Licorice self-assembled nanoparticles (LD-SANs) were prepared by mixing water and ethanol extracts of licorice, followed by ethanol evaporation under reduced pressure to trigger nanoprecipitation. In vitro transdermal tests compared the delivery efficiency of six major bioactive compounds between LD-SANs and traditional licorice decoction (LD). The penetration mechanism was investigated via passive diffusion and cellular uptake studies. In an AD mouse model, the therapeutic effects and expression of tight junction (TJ) proteins (Occludin and Claudin-1) were assessed. **Results:** The average particle size of LD-SANs is 200 nm, and it is uniform and stable. LD-SANs significantly enhanced the delivery efficiency of all six bioactive compounds compared to LD. Mechanistic studies revealed a unique “dual-channel” penetration mechanism: the nanoscale size enabled passive diffusion through hair follicles, intercorneocyte lipid gaps, and skin appendages, while perifollicular antigen-presenting cells (APCs) actively recognized and internalized the nanoparticles, creating a cell-mediated active targeting route that collectively boosted skin accumulation. In the AD model, LD-SANs promoted the expression of Occludin and Claudin-1 in the epidermal granular layer, reinforcing intercellular barrier integrity. **Conclusions:** By combining “efficient penetration” and “barrier repair”, LD-SANs demonstrated notable therapeutic efficacy in AD. This work transforms a traditional decoction into a well-characterized, high-performance nanomedicine and offers a novel strategy for developing TCM-based transdermal delivery systems.

## 1. Introduction

Transdermal drug delivery, with its unique advantages of enabling localized targeted delivery, enhancing therapeutic precision, and reducing systemic exposure risk, has garnered considerable attention in the field of dermatological treatment. The core strategy for improving transdermal delivery efficiency centers on overcoming the stratum corneum (SC) barrier [1,2]. Conventional formulations, such as free drugs or those simply supplemented with chemical penetration enhancers, often exhibit limited transdermal efficiency constrained by the drug’s physicochemical properties (e.g., lipophilicity, molecular size) and skin barrier function, resulting in low bioavailability and difficulty maintaining effective concentrations at the target site [3,4]. To address this, various nanocarriers (e.g., liposomes, polymeric nanoparticles, and microemulsions) have been extensively developed. They enhance skin retention, enable controlled release, and leverage their nano-size for passive penetration via multiple pathways such as hair follicles and intercellular spaces, significantly improving the delivery efficiency of hydrophobic drugs and macromolecules [5,6,7]. However, existing nano-systems still face several challenges: synthetic carriers may raise concerns regarding biocompatibility and long-term safety, their loading process might disrupt the inherent ratio and synergistic effects of multi-component herbal systems, and complex preparation processes hinder industrial-scale production [8].

In recent years, self-assembled nanoparticles (SANs) spontaneously formed during the decoction of traditional Chinese herbal formulas have emerged as a highly promising research direction in nanomedicine [9,10]. Typically composed of natural bioactive components like polysaccharides, saponins, and flavonoids, these SANs not only boast a natural origin but may also preserve the synergistic therapeutic effects of the original formula components, making them attractive candidate carriers [11,12]. However, the assembly process of these nanostructures, formed under mild aqueous conditions, is often difficult to control precisely. Moreover, they suffer from poor long-term physical stability in aqueous environments, being prone to Ostwald ripening or aggregation, which leads to the loss of their nano-size advantages and severely impacts the reproducibility, transdermal performance, and ultimate drug development potential of the formulations [13,14]. Consequently, developing a nano-delivery platform that combines natural origin, high stability, and a simple preparation process has become an urgent need for advancing the modernization of transdermal herbal formulations.

To address the aforementioned challenges, constructing a transdermal nano-platform capable of overcoming the stability bottleneck while fully preserving the synergistic advantages of herbal multi-components is crucial. Licorice (*Glycyrrhiza* spp.), a classic traditional Chinese herb, has a long history of use in treating inflammatory skin diseases. Modern pharmacological studies have shown that its rich content of flavonoids and triterpenoid saponins possesses significant anti-inflammatory activity and can promote the expression of key epidermal barrier proteins (e.g., Occludin, Claudin-1), demonstrating dual potential for “anti-inflammation” and “barrier repair” [15,16,17]. This makes it an ideal model for constructing functional nano-delivery systems. Atopic dermatitis (AD), a chronic, relapsing inflammatory skin disease, is characterized pathologically by a vicious cycle where “immune-inflammatory dysregulation” and “skin physical barrier defect” exacerbate each other [18,19]. Therefore, an ideal therapeutic strategy for AD must simultaneously address these two core aspects. Although the traditional licorice decoction contains the aforementioned active components, it struggles to effectively penetrate intact or compromised skin barriers. We hypothesize that upgrading the licorice decoction into a stable, uniform nano-formulation could not only significantly enhance the transdermal delivery efficiency of its active components but might also, due to its nano-properties, give rise to a novel delivery mechanism. Beyond size-dependent passive targeting and penetration, the nanoparticles might be able to interact with the cutaneous immune microenvironment (e.g., highly active antigen-presenting cells (APCs) surrounding hair follicles), forming a unique active targeting process. This interaction with the immune system could help create more effective penetration pathways, subsequently activating the expression and functional remodeling of key proteins in the skin barrier, ultimately achieving synergistic repair of AD skin lesions. However, key scientific questions remain unresolved: how to precisely control the self-assembly process of licorice components to prepare stable, uniform nanoparticles, and how to systematically verify their proposed “passive–active” dual-channel transdermal mechanism and their dual therapeutic effects on AD.

Several methods have been reported for preparing SANs from natural products, including direct decoction-induced self-assembly, solvent exchange, pH-driven assembly, and micro-precipitation methods [20,21,22]. Among these, the micro-precipitation method offers distinct advantages in controlling particle size, improving batch-to-batch reproducibility, and avoiding toxic organic solvents. In this study, we therefore adopted a modified micro-precipitation method approach to enhance the stability and uniformity of licorice-derived nanoparticles, which is more suitable for transdermal delivery and industrial translation than traditional decoction-based assembly. Based on this, using licorice as the model herb, this study aims to prepare high-performance licorice-derived self-assembled nanoparticles (LD-SANs) by precisely regulating the controlled precipitation process of its water-extracted and alcohol-extracted components, thereby addressing the issues of poor stability and size heterogeneity associated with the traditional licorice decoction (LD). This study systematically evaluates the physicochemical properties, stability, and enhancing effect of LD-SANs on the transdermal penetration of major licorice active components. Furthermore, it delves into the transdermal mechanism, focusing on verifying the hypothesized dual-channel delivery behavior combining “passive diffusion and cell-mediated active targeting.” Finally, the dual therapeutic efficacy of “anti-inflammation” and “barrier repair” achieved by LD-SANs via this efficient delivery system is comprehensively evaluated in an AD mouse model. This study not only aims to transform a traditional decoction into a high-performance nanomedicine with a clarified mechanism, but it also provides a new strategy and theoretical basis for developing intelligent transdermal delivery systems based on Traditional Chinese Medicine (TCM).

## 2. Materials and Methods

### 2.1. Reagents

Reference standards of Apioglycyrrhizin (AG), Glycyrrhizin (GL), and Isoglycyrrhizin (IG) (purity > 98.0%) were purchased from Push Bio-technology Co., Ltd. (Chengdu, China). Apioglycyrrhetinic acid glycoside (AGly), Glycyrrhizic acid (GA), and Licochalcone A (LicA) (purity > 98.0%) were obtained from Chenguang Biotechnology Co., Ltd. (Baoji, China). Licorice was supplied by Qiancao Traditional Chinese Medicine Pieces Co., Ltd. (Beijing, China). The ACQ (aggregation-induced quenching) probe (P4) was kindly provided by Prof. Wei Wu’s research group at Fudan University (Shanghai, China), the molecular structure of P4 is shown in Figure S2. Coumarin 6 (C6, purity > 98.0%) was purchased from Macklin Biochemical Co., Ltd. (Shanghai, China). The Langerin/CD207, Occludin, and Claudin-1 antibodies were acquired from Thermo Fisher Scientific (Waltham, MA, USA). 2,4-Dinitrochlorobenzene was obtained from Sigma-Aldrich (St. Louis, MI, USA). ELISA kits for IL-6 and IgE were supplied by Elabscience Biotechnology Inc. (Wuhan, China). PrimeScript™ RT Master Mix was purchased from Takara (Kyoto, Japan), and 2×T5 Fast qPCR Mix (SYBR Green I) was acquired from Tsingke Biotechnology Co., Ltd. (Beijing, China).

### 2.2. Animals

Male SD rats (180–220 g) and male BALB/c mice (18–22 g), both of specific pathogen-free (SPF) grade, were purchased from SPF (Beijing) Biotechnology Co., Ltd. (Beijing, China).

### 2.3. Fabrication of Nanoparticles

LD was prepared according to the traditional method. Briefly, 100 g of licorice pieces was immersed in 800 mL of distilled water for 30 min, followed by reflux extraction for 1 h. The residue was subjected to a second extraction under the same conditions. The combined filtrates were concentrated using a rotary evaporator at 60 °C under reduced pressure to a final concentration of 0.2 g crude drug/mL (referred to as LD).

LD-SANs were fabricated using a modified micro-precipitation method based on our previously reported protocol with optimization [23]. Briefly, after the aqueous extraction described above, the residue was further extracted with 600 mL of 70% ethanol under reflux for 1 h to obtain the ethanol extract. The aqueous extract and the ethanol extract were combined and stirred at 600 rpm for 20 min at room temperature. The mixture was then concentrated under reduced pressure using a rotary evaporator at 60 °C to remove ethanol and achieve a final concentration of 0.2 g crude drug/mL. In this process, the volatilization of the organic phase gradually reduces the dissolving capacity of the mixed solvent, inducing homogeneous nucleation of the alcohol-soluble components under low supersaturation, while surface-active substances in the water extract inhibit particle growth and aggregation.

Fluorescently labeled nanoparticles were prepared by adding coumarin-6 (C6, 25 μg/mL) and the aggregation-induced quenching probe P4 (5 μg/mL) to the combined filtrates prior to concentration. The mixture was protected from light during subsequent concentration steps to obtain fluorescently labeled LD and LD-SANs at 0.2 g crude drug/mL.

### 2.4. Particle Size, Polydispersity Index (PDI), and Zeta Potential Determination

Key parameters (particle size, PDI, and zeta potential) of LD and LD-SANs were measured in triplicate at 25 °C using a nanolaser particle size analyzer (Nanotrac Wave II, Microtrac, York, PA, USA) after 10-fold dilution with deionized water.

### 2.5. Stability Testing of LD and LD-SANs

The formulations were stored at 4 °C in hermetically sealed Eppendorf tubes (EP tubes), and their stability was assessed throughout a 7-day period via daily visual inspection and measurements of particle size and PDI (in triplicate at 25 °C using a nanoparticle size analyzer).

### 2.6. Encapsulation Efficiency (EE) and Drug Loading Capacity (DL) of LD and LD-SANs

The EE of LD and LD-SANs for six main components (AG, GL, AGly, IG, GA, and LicA) was determined by an ultrafiltration–centrifugation method. Briefly, 5 mL of each sample was centrifuged at 3000 r·min^−1^ for 30 min to collect the precipitate. The supernatant was subsequently transferred to 3000 Da ultrafiltration tubes and centrifuged at 13,000 r·min^−1^ for another 30 min. The nanoparticle fraction and the true solution were collected from the bottom and upper layers, respectively. All samples were analyzed by HPLC. The EE and DL of LD and LD-SANs were then calculated. Here, W_t1_, W_t2_, and W_t_ represent the content of the active ingredient in the nanoparticle fraction, in the total LD or LD-SANs formulation, and in the licorice herb, respectively.

$$EE\%=W_{t1}/W_{t2}\times 100\%\;\;DL\%=W_{t2}/W_{t}\times 100\%$$

### 2.7. Characterization of LD-SANs

We first sought to characterize the basic physicochemical properties of the fabricated SANs. Freeze-dried samples of LD, alcohol extraction, and LD-SANs were characterized by UV-Vis (200–800 nm), UV-240IPC (Shimadzu, Japan), FT-IR (4000–400 cm^−1^, IRTracer 100, Shimadzu, Japan), and XRD (10–90° 2θ, Cu-Kα radiation, D8 ADVANCE, Bruker, Germany). Thermal properties were analyzed by DSC (25–350 °C, 10 °C/min under N_2_, Hitachi DSC200) and TGA (25–600 °C, 10 °C/min under N_2_, NETZSCH STA449F5); approximately 10 mg of each sample was used for each thermal analysis measurement. The surface morphology of LD and LD-SANs was characterized by scanning electron microscopy (SEM, KYKY Technology Co., Ltd., Beijing, China). Prior to observation, the samples were sputter-coated with a thin gold film for electrical conduction. SEM images were captured at an accelerating voltage of 5 kV under high-vacuum conditions.

### 2.8. Fluorescence Stability of LD and LD-SANs

The stability of LD and LD-SANs was evaluated by monitoring the fluorescence intensity of probes P4 and C6 in phosphate buffer (pH 6.8) and 1% Tween 80 (*w*/*v*) solution. Specifically, 1 mL of each sample was added to 20 mL of each medium, thoroughly mixed, and incubated at 32 °C for 24 h. Aliquots (1 mL) were collected at 0, 2, 4, 8, 12, and 24 h for fluorescence measurement using a spectrophotometer (RF-6000, Shimadzu, Japan). The detection parameters were set as follows: for P4, Ex/Em were 640/630–700 nm, for C6, Ex/Em were 465/450–550 nm, with both measured at a scanning speed of 2000 nm/min.

### 2.9. Franz Diffusion Cell In Vitro Skin Permeability Test

Franz diffusion cells (TP-6, Xinzhou Technology, Shanghai, China) were used to study the in vitro permeation of LD and LD-SANs. Rat abdominal skin was prepared by removing subcutaneous fat and mounted between compartments (SC facing donor). The receptor phase was PBS (pH 7.4) with 1% Tween 80. The donor phase contained 1 mL of SANs. Under conditions of 32 °C and 300 rpm stirring, 1 mL samples were collected from the receptor at predetermined time points (6, 12, 18, 24, 30, 36, 42, and 48 h) and replaced with fresh medium. After 48 h, the skin was rinsed with ethanol and extracted with methanol via ultrasonication.

### 2.10. HPLC Analysis

HPLC analysis for quantifying active licorice components was performed on an Inertsustain-C18 column (4.6 mm × 250 mm, 5 μm). The mobile phase, comprising (A) 0.1% aqueous phosphoric acid and (B) acetonitrile, was delivered at 1 mL/min. The column temperature and injection volume were set at 30 °C and 10 μL, respectively. The mobile phase was filtered and sonicated before use.

### 2.11. SEM and H&E Staining of Skin

Skin samples treated with normal saline, LD, or LD-SANs via transdermal administration for 48 h were prepared using the same SEM protocol. Briefly, treated skin tissues were dehydrated via a graded ethanol series and lyophilized, and then mounted on double-sided carbon tape fixed to aluminum stubs. All tissue specimens were sputter-coated with a thin gold layer before observation, and SEM imaging was performed at an accelerating voltage of 5 kV under high-vacuum conditions.

Skin samples were immediately fixed in 4% paraformaldehyde. The fixed tissues were then embedded in paraffin and sectioned into 3–4 μm thick slices. Histological analysis was performed using hematoxylin and eosin (H&E) staining.

### 2.12. FTIR and DSC Analysis of Skin

The skin samples were removed, rinsed with distilled water, and prefrozen at −80 °C for 1 h. After freeze-drying for 48 h, the samples were cut into small pieces. For DSC analysis, 4 mg samples were placed on an aluminum plate and scanned from 30 to 400 °C at a rate of 5 °C/min. For FTIR analysis, the prepared intact skin tissue pieces were directly determined using ATR-FTIR mode over the wavenumber range of 400–4000 cm^−1^, with a resolution of 2 cm^−1^ and 100 cumulative scans.

### 2.13. In Vivo Skin Permeability Test

SD rats were depilated 24 h prior to the experiment. Under anesthesia induced by sodium pentobarbital (30 mg/kg), the abdominal skin was affixed with a Franz diffusion cell (effective area: 1.77 cm^2^). The donor compartment received 1 mL of fluorescently labeled LD or LD-SANs solution and was light-protected with aluminum foil. At designated time points (1, 2, 4, 6, 8, and 12 h), the rats were euthanized, and the exposed skin was collected, saline-rinsed, snap-frozen in liquid nitrogen, and stored at −80 °C.

### 2.14. Vertical and Horizontal Sections of the Skin

The skin samples were embedded in OCT compound and rapidly frozen in liquid nitrogen or stored at −80 °C. Each skin sample was divided into two parts, which were then sectioned vertically and horizontally, respectively. Serial horizontal sections were cut at 10 μm intervals. Every fourth section was collected for subsequent DAPI or Immunofluorescence (IF) staining.

### 2.15. IF Staining

Following antigen retrieval, frozen skin sections were washed twice with PBS and blocked with donkey serum for 1 h. Sections were then incubated with primary antibody overnight at 4 °C. After PBS washing and treatment with 0.5% Tween 20 for 20 min, a signal enhancer was applied for 30 min. This was followed by incubation with anti-CD207 antibody for 4 h at room temperature and donkey anti-mouse IgG H&L for 1 h. Finally, sections were mounted with an anti-fade medium to minimize bubbles.

### 2.16. Confocal Laser Scanning Microscope (CLSM) Observation

Confocal laser scanning microscopy (CLSM) (LSM510, Carl Zeiss, Jena, Germany) was employed to examine the prepared sections. The sections were scanned at a magnification of 10×. DAPI and C6 were excited using their respective default channels. IF signals were excited via the A405 channel. P4 signals were specifically excited using the Alexa 633 channel.

### 2.17. Modeling of 2,4-Dinitrochlorobenzene (DNCB)-Induced AD

Forty BALB/c mice were randomized into four groups: control, model, Crisaborole, and LD-SANs. Except for the control group, mice were sensitized with 1% DNCB (200 µL) on days 1 and 3, and then challenged with 0.5% DNCB every other day. The treatments were applied daily for 10 days from day 5 onward, with skin conditions monitored by photography and EASI scoring. Upon termination, the body weights were recorded and the mice were euthanized. Blood serum was stored at −80 °C, skin samples were fixed in 4% paraformaldehyde (PFA), and spleens were weighed to calculate the splenic index.

### 2.18. Enzyme-Linked Immunosorbent Assay (ELISA)

The concentrations of IL-6 and IgE cytokines in animal serum were measured according to the kit instructions, with a detection sensitivity of <5 pg/mL.

### 2.19. Immunohistochemical Staining

Mouse dorsal skin paraffin sections were processed for immunohistochemistry using primary antibodies against Occludin and Claudin-1. After overnight incubation at 4 °C and PBS washing, sections were treated with HRP-conjugated anti-rabbit IgG (ZSGB-BIO) for 30 min at room temperature. Following additional washes, antibody binding was detected using 3,3′-diaminobenzidine (DAB) as the substrate.

### 2.20. RT-qPCR

Total RNA was extracted from skin tissue using Trizol reagent. According to the kit instructions, reverse transcription (RT) was performed with a reverse transcription kit to obtain cDNA. Subsequently, specific primers were used to amplify the variable and constant regions of the following genes: *Occludin* (Forward: CCAATGTCGAGGAGTGGG; Reverse: CGCTGCTGTAACGAGGCT); *Claudin-1* (Forward: ATGAGGTGCAGAAGATGAGG; Reverse: GGTGTTGGGTAAGAGGTTGT); *IL-1β* (Forward: GCTTCAAATCTCGCAGCAGC; Reverse: TCACAGAGGATGGGCTCTTC); *GAPDH* (Forward: TGAAGGGTGGAGCCAAAAGG; Reverse: AAGGTGGAAGAGTGGGAGTT); and *IL-6* (Forward: TACATCCTCGACGGCATCTC; Reverse: TTTCAGCCATCTTTGGAAGG).

### 2.21. Statistical Analysis

All results were expressed as mean ± standard deviation (SD). The statistical significance was as follows: *p* < 0.05: statistically significant; *p* < 0.01: considered very significant. Parameters such as in vitro transdermal penetration, formulation characterization, and stability of the fluorescent medium were determined by the use of Origin 2021 (OriginLab Corporation, Northampton, MA, USA). The analysis and computation of the pharmacodynamic results were performed with GraphPad Prism 6.02 (GraphPad Software, Inc., La Jolla, CA, USA).

## 3. Results

### 3.1. Preparation of SANs

**Figure 1:**
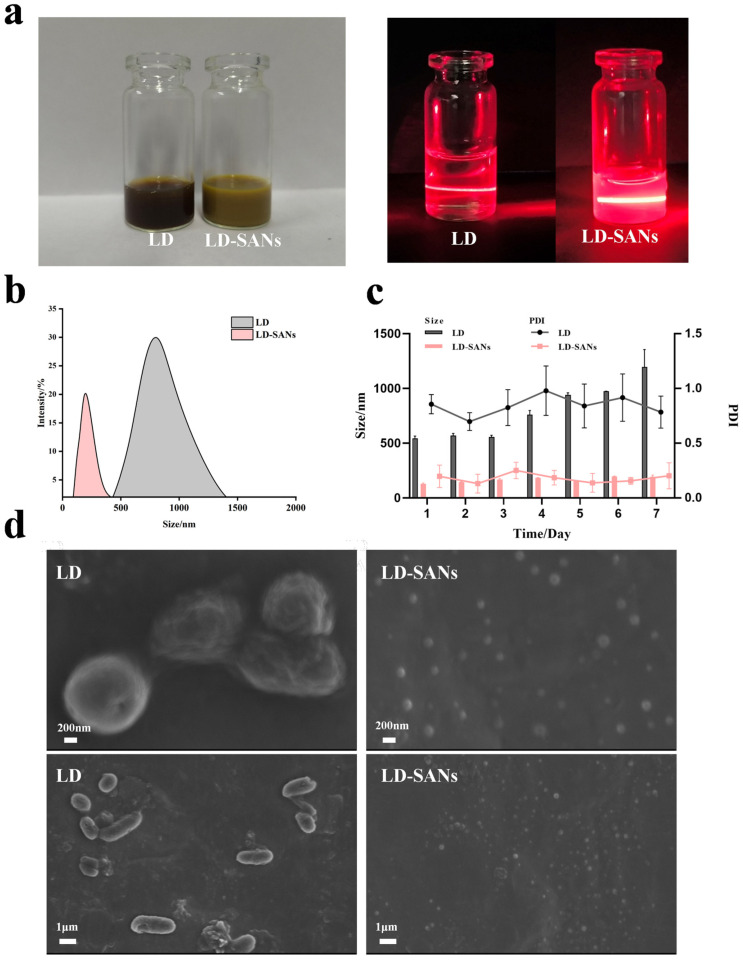
Schematic illustration of the preparation process and characterization of LD-SANs. (**a**) Schematic diagram of the controlled micro-precipitation method for LD-SANs fabrication. (**b**) Appearance of LD and LD-SANs suspensions. (**c**) Size distribution of LD and LD-SANs measured by DLS. (**d**) SEM images of LD and LD-SANs. Scale bar: 200 nm/1 μm.

LD and LD-SANs appeared as reddish-brown and brown–yellow suspensions, respectively (Figure 1a). The formation of LD-SANs was achieved through a controlled micro-precipitation mechanism. In this process, the aqueous extract (containing amphiphilic saponins, polysaccharides, and proteins) served as the poor solvent system, while the ethanol extract (enriched with hydrophobic flavonoids) acted as the good solvent. Upon mixing and subsequent ethanol evaporation under reduced pressure at elevated temperature (60 °C), the solubility of hydrophobic components decreased sharply, triggering their rapid precipitation. Concurrently, the amphiphilic components facilitated the self-assembly process through non-covalent interactions (hydrogen bonding, hydrophobic interactions, and π-π stacking), resulting in the formation of uniform nanoparticles with a hydrophobic core (enriched with flavonoids) and a hydrophilic shell (enriched with saponins and polysaccharides).

Dynamic light scattering (DLS) analysis revealed that LD exhibited submicron-sized particles with a polydispersity index (PDI) greater than 0.3 and a slightly negative zeta potential, indicating a broad size distribution and tendency toward aggregation (Figure 1b, Table S1). PDI reflects the particle size distribution homogeneity of nanosystems. A smaller PDI indicates a narrower size distribution and better dispersion stability. Generally, PDI < 0.2 is the optimal range for uniform nanoparticles, and PDI below 0.3 is acceptable. A PDI higher than 0.3 implies a broad size distribution and a tendency toward particle aggregation. In contrast, LD-SANs prepared under optimized conditions showed a hydrodynamic diameter of 189.5 ± 0.3 nm, a narrow PDI of 0.138 ± 0.130, and a highly negative zeta potential of −31.4 ± 0.8 mV (Figure 1b, Table S1). The significantly more negative zeta potential of LD-SANs compared to LD (−31.4 mV vs. −12.6 mV) indicates enhanced electrostatic stabilization, which contributes to their improved colloidal stability. The Tyndall effect was more pronounced in LD-SANs, reflecting a higher concentration of uniformly dispersed nanoparticles.

Scanning electron microscopy (SEM) imaging confirmed the morphological differences between the two formulations (Figure 1d). LD-SANs appeared as homogeneous spherical nanoparticles with smooth surfaces and an average diameter of approximately 200 nm, consistent with DLS measurements. In contrast, LD exhibited irregular microparticles with rough surfaces and a low density of nanoparticles, indicating incomplete or uncontrolled self-assembly during traditional decoction preparation.

These results demonstrate that the controlled precipitation of the ethanol extract (organic phase) into the aqueous extract (inorganic phase) effectively addresses the limitations of conventional SANs derived from traditional decoctions, yielding uniform, stable nanostructures with desirable physicochemical properties for transdermal delivery applications.

### 3.2. Stability of LD and LD-SANs

As demonstrated in Figure 1c, LD had larger particle sizes and PDI values than LD-SANs. It is important to note that LD-SANs were stable with respect to their original particle size and low PDI during the one-week period of study. On the other hand, the LD started exhibiting a gradual particle size growth starting on day 4, which signifies the start of aggregation. All of these results prove that LD-SANs possess not only a smaller particle size but also a more stable and robust nanostructure in comparison with LD (Table S2).

The fluorescence stability of P4 and C6 within both types of SANs was evaluated in phosphate buffer (pH 6.8) and PBS containing 1% Tween 80 at 32 °C (Figure S3). In the pH 6.8 buffer, after 12 h, C6 retained 79.86 ± 4% of its initial fluorescence in LD and 90.6 ± 0.58% in LD-SANs (Figure S3b,d), while P4 retained 89.09 ± 2.06% and 80.56 ± 0.87% (Figure S3a,c), respectively. In PBS with 1% Tween 80, both fluorophores remained highly stable, with approximately 99.47 ± 0.57% retention for C6 and 108.9 ± 0.12% for P4 in LD-SANs. These findings confirm that C6 and P4 are highly stable in both LD and LD-SANs under mildly acidic conditions (pH 6.8) at skin temperature (32 °C), indicating their suitability for dermal applications.

### 3.3. EE and DL of LD and LD-SANs

Phase separation analysis of LD and LD-SANs showed that the fraction of nanoparticles had the greatest concentration of active ingredients in both formulations, which showed that most of the active compounds were encapsulated in the nanoparticles. These nanoparticles likely serve as the primary material basis for their effectiveness (Tables S3 and S4). Moreover, the nanoparticle fraction of LD-SANs was higher than the active ingredients of LD (Table 1). AG, GL, IG, and GC were the main active components that were found in over 80%, reaching 90.25%, 86.67%, 91.79%, and 91.59%, respectively, which are 1.6, 1.57, 1.64, and 1.65 times higher than in LD. The AGly and LicA fractions in the nanoparticle fraction of LD-SANs were 75.91% and 55.48% of LD, respectively, which is increased 1.73 and 1.28 times, respectively. These results show that LD-SANs are more efficient in the encapsulation of licorice active components during the self-assembly procedure to create nanoparticles compared to LD.

The regression equations, precision, repeatability, stability, and recovery of the active components in LD-SANs were validated, with the corresponding results presented in Figure S1 and Tables S6–S9. the HPLC analysis found that the drug loading (DL) of active components in LD-SANs was greater than in LD (Table 2), with a significant increase in the DL of small-molecule flavonoid compounds. The DL values of AG, GL, and IG in LD-SANs were 82.38%, 73.92%, and 60.86%, respectively, which are 1.09, 1.19, and 1.69 times higher than LD. The DL of LicA was 48.68%, and is 6.9 times greater than in LD. These results indicate that, compared to LD, LD-SANs can significantly enhance the drug loading of multiple active components derived from licorice.

### 3.4. Physicochemical Properties of LD-SANs

**Figure 2:**
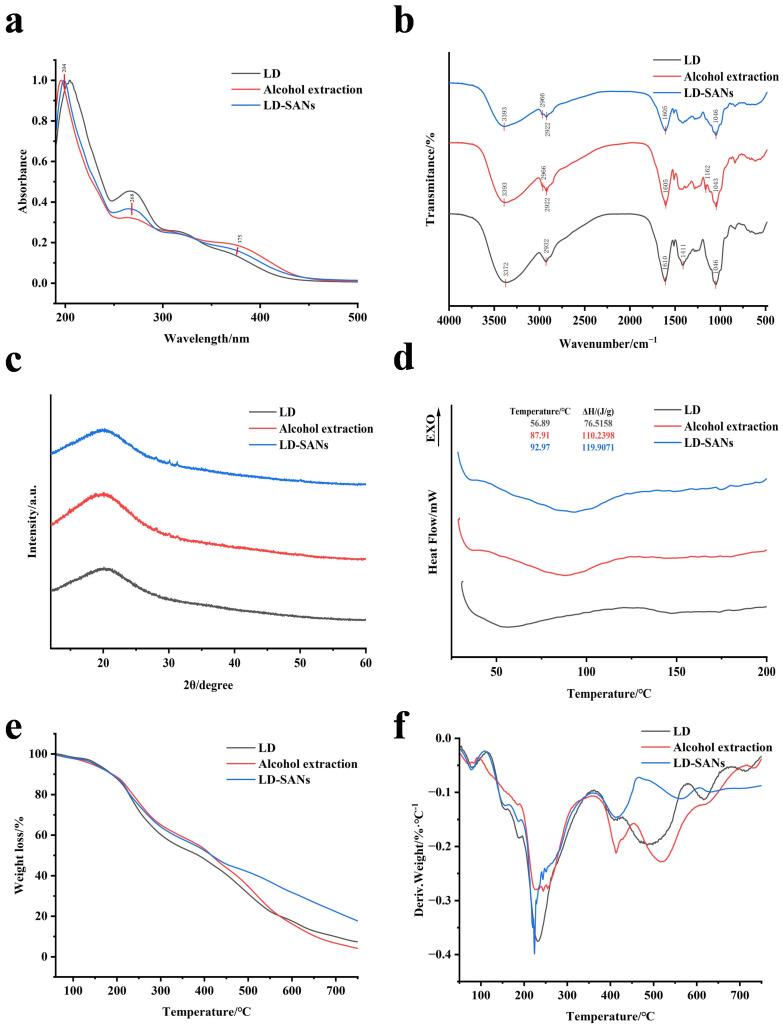
Characterization of LD, alcohol extraction, and LD-SANs. (**a**) The UV spectrophotometric analysis of LD, alcohol extraction, and LD-SANs. (**b**) The FT-IR spectra of LD, alcohol extraction, and LD-SANs. (**c**) The XRD analysis of LD, alcohol extraction, and LD-SANs. (**d**) The DSC analysis of LD, alcohol extraction, and LD-SANs. (**e**) The TGA thermogram of LD, alcohol extraction, and LD-SANs. (**f**) The DTG curve of LD, alcohol extraction, and LD-SANs.

UV-Vis analysis of LD-SANs indicated absorption peaks at 204, 268, and 375 nm, which were shifted compared to those of the individual LD as well as alcohol extraction (Figure 2a). The increased absorbance at 268 nm relative to the alcohol extraction at 375 nm relative to LD indicates intermolecular interaction influencing the conjugated electron system.

FT-IR spectra (Figure 2b) showed no evidence of new covalent bonds in LD-SANs. However, peak shifts were observed: O–H stretching (3393 to 3372 cm^−1^), saturated C–H stretching (2922 to 2932 cm^−1^), and C–H stretching (1605 to 1610 cm^−1^). Intensity increases at 1608 and 1046 cm^−1^, the disappearance of the benzene ring C–H peak at 2966 cm^−1^, and the appearance of a new C–H bending vibration at 1411 cm^−1^ suggest a non-covalent interaction, which is likely important for the self-assembly of nanoparticles.

XRD patterns (Figure 2c) exhibited a broad peak around 20° for all samples, with slightly higher intensity found in LD-SANs alongside the alcohol extraction, which indicates an amorphous structure with improved molecular organization.

DSC analysis (Figure 2d) showed that three samples exhibited a major endothermic peak during the heating process, showing a trend of “increasing peak temperature and greater enthalpy change.” Compared to LD and alcohol extraction, LD-SANs had the highest phase transition temperature (92.97 °C) and the greatest enthalpy change (119.9071 J/g), demonstrating the strongest thermal stability.

The TGA results (Figure 2e,f) revealed that the main weight loss for LD-SANs began at 106 °C, which is higher than the 91 °C onset observed for the alcohol extraction, and that LD-SANs decomposed more slowly than LD. Furthermore, LD-SANs exhibited less overall mass loss after 430 °C, indicating improved thermal stability, likely because of more ordered molecular packing following self-assembly. We compared the thermal decomposition behavior of LD-SANs with that of other polysaccharide/saponin-based nanoparticles and determined that our results are consistent with previous reports on polysaccharide-based nanoparticles [24].

### 3.5. In Vitro Skin Permeation Study

**Figure 3:**
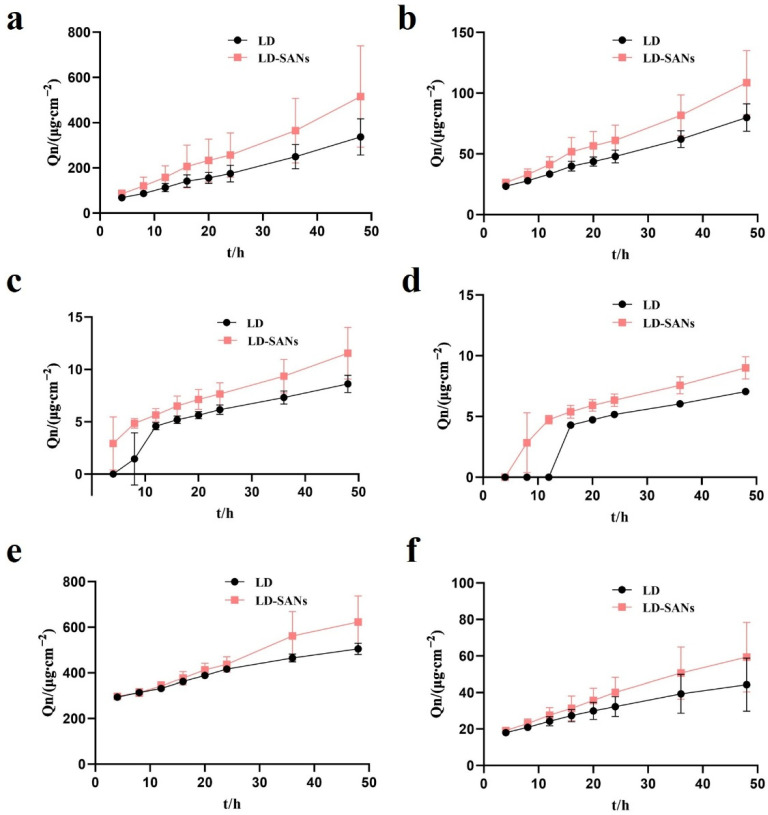
Transdermal characterization of LD and LD-SANs in vitro. (**a**–**f**) The 48 h cumulative penetration Qn of AG, GL, AGly, IG, GA, and LicA in the skin. All data are presented as the mean ± SD, *n* = 3.

The transdermal penetration of LD and LD-SANs was evaluated by measuring the cumulative permeation (Qn), skin retention (Qs), permeation rate (Js), and permeability coefficient (Kp) (Table 3 and Figure 3). After 4 h, the Qn values for AG, GL, AGly, and IG in the LD-SANs group began to exceed those in the LD group. At 48 h, the Qn values reached 516.2 ± 223.64, 108.72 ± 26.33, 11.56 ± 2.46, and 9.01 ± 0.92 μg/cm^−2^, which were approximately 1.53, 1.36, 1.34, and 1.27 times higher, respectively, than those observed with LD. Similarly, the Qn values for GA and LicA in the LD-SANs group were significantly greater at 24 h, reaching 557.42 ± 85.93 and 59.39 ± 19.02 μg/cm^2^ at 48 h—about 1.10 and 1.34 times higher than in the LD group. These findings indicate that LD-SANs substantially enhance the transdermal delivery of active compounds derived from licorice.

The Qn-t curves were fitted to different release models (Table S5), and the resultant values of Qs and Js are given in Table 3. All components in LD-SANs had a higher permeation rate (Js) than in LD, which is indicative of improved penetration of the SC into more profound skin layers. GL and LicA showed a significant difference in skin retention (Qs), with the latter having a considerably larger accumulation in the LD-SANs group. This implies an increase in the volume of reservoirs in the skin tissues, which may increase anti-inflammatory and antibacterial effects. All other components of the SANs except IG exhibited good fits (R^2^ > 0.8) to zero-order, first-order, Higuchi, and Ritger–Peppas models, which suggested sustained and controlled transdermal delivery.

### 3.6. Analysis of Penetration Mechanism

**Figure 4:**
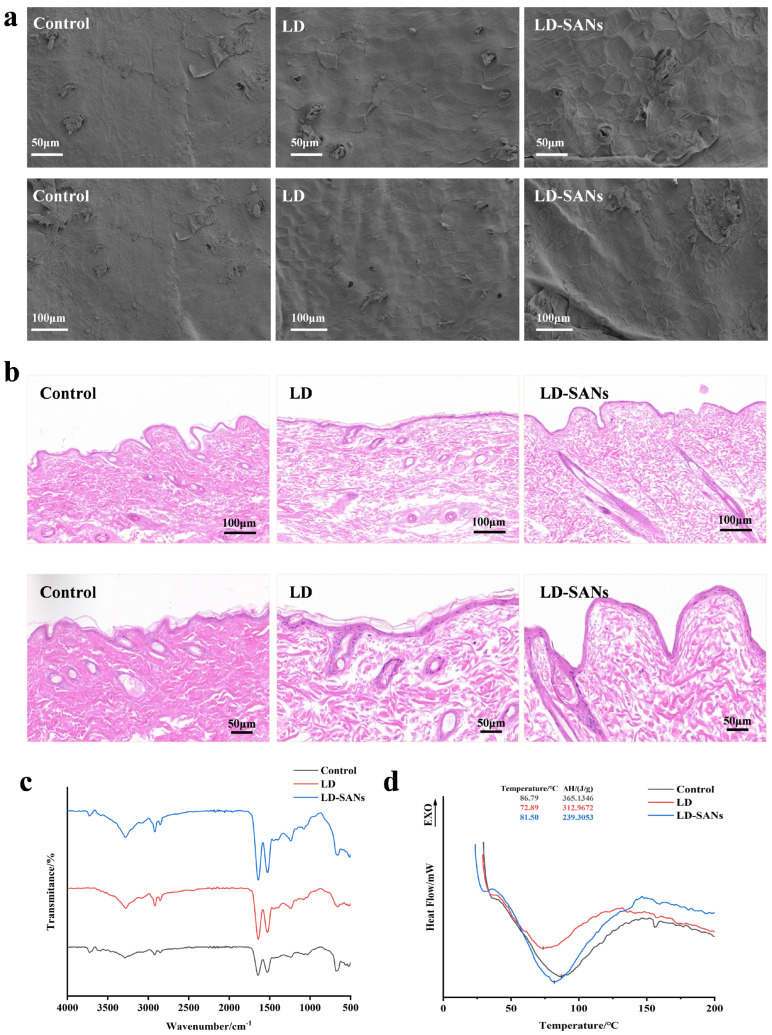
Microstructure of the rat skin and histopathological microscopic slides. (**a**) The SEM images of skin surfaces from in vitro transdermal experiments; scale bar: 50 μm/100 μm. (**b**) HE staining of skin longitudinal sections from in vitro transdermal experiments; scale bar: 50 μm/100 μm. (**c**) FTIR spectra of the SC treated with the control, LD, and LD-SANs. (**d**) DSC thermograms of skin treated with the control, LD, and LD-SANs.

SEM analysis of the treated rat skin showed that the surface of the control rat skin had a smooth and intact SC with limited surface roughness and no attached substances (Figure 4a). However, the surface of the skin exposed to LD and LD-SANs showed a honeycomb-like structure with SC curled and hair follicle opening dilation.

H&E staining (Figure 4b) revealed that the control rat skin had well-organized layers with an intact epidermis and SC, and the basal layer had tightly stacked cells. Conversely, LD made the SC loose and thin, and LD-SANs caused the cuticle to further fall off the skin.

We further investigated the effects of nanoparticles on cuticle protein and keratin of the skin through DSC and FTIR detection. The characteristic peaks of SC lipids within the C-H stretching vibration region (2800–3000 cm^−1^) of infrared spectra serve as indicators of lipid structural organization and order (Figure 4c). Specifically, the peaks observed at 2920–2930 cm^−1^ and 2850–2860 cm^−1^ correspond to the asymmetric and symmetric stretching vibrations of lipid CH_2_ groups, respectively. Compared to the control group, treatment with SANs induced a rightward shift in the CH_2_ asymmetric stretching vibration peak, indicating a decrease in lipid orderliness. Hydration characteristics of the SC are evidenced by strong absorption in the amino acid region (1500–1700 cm^−1^) and a broad water absorption band (3000–3600 cm^−1^). Notably, the amide I band (1600–1700 cm^−1^) and amide II band (1500–1600 cm^−1^), which arise from keratin vibrational signals, exhibited redshifts from 1643 cm^−1^ to 1641 cm^−1^ and from 1529 cm^−1^ to 1527 cm^−1^, respectively. This redshift implies a conformational transition of keratin from an alpha-helix to a random coil structure. The symmetrical stretching vibration frequency of O-H groups, located between 3200 and 3300 cm^−1^, produces a broad absorption peak attributable to hydrogen bonding among water molecules, thereby directly reflecting the moisture content of the SC. Comparative analysis reveals that the absorption peak intensity near 3300 cm^−1^ in the control group is markedly lower than that observed in the two SANs-treated groups, indicating a significant enhancement of SC hydration following SANs treatment. Collectively, the observed decrease in lipid structural order and the concomitant increase in hydration contribute to weakening the barrier function of the SC, thereby facilitating enhanced transdermal drug penetration.

Thermograms corresponding to reference data from rat skin treated with the control, LD, and LD-SANs are shown in Figure 4d. Between 65 °C and 120 °C, a relatively broad endothermic region is observed. The control group exhibits the highest peak temperature (86.79 °C) and enthalpy change (365.13 J/g), indicating the most stable lipid structure. In contrast, although the LD-treated group has the lowest peak temperature, its enthalpy change remains close to that of the control group. The LD-SANs-treated group exhibits a significantly reduced enthalpy change (239.31 J/g), indicating the poorest crystallization ability and thermal stability. This is likely due to the disruption of the ordered arrangement of lipid molecules by nanoparticles, accompanied by keratin denaturation.

### 3.7. In Vivo Transdermal Penetration Monitored by Dual Fluorescent Probes

To investigate the transdermal properties of the SANs, the near-infrared fluorescent probe P4 was used to track the in vivo fate of the SANs [25]. A notable feature of the P4 probe is its sensitive aggregation-caused quenching (ACQ) effect through π-π stacking upon contact with water. Since the skin regulates water loss, with water content reaching 75% in the epidermis and even the relatively dry subcutaneous skin containing about 15% water [26], this switchable signal provides an accurate and sensitive means to track intact SANs in the skin [25]. Meanwhile, coumarin C6, a common fluorescent dye with high sensitivity and selectivity, is widely used to develop drug delivery systems, label drug carriers, and study drug distribution, metabolism, and release processes in vivo [27,28]. C6 can label cargo molecules as effectively encapsulated within SANs, simulating the diffusion behavior of free molecules released by nanoparticles in vivo [29].

#### 3.7.1. Analysis of Vertical Skin Sections

**Figure 5:**
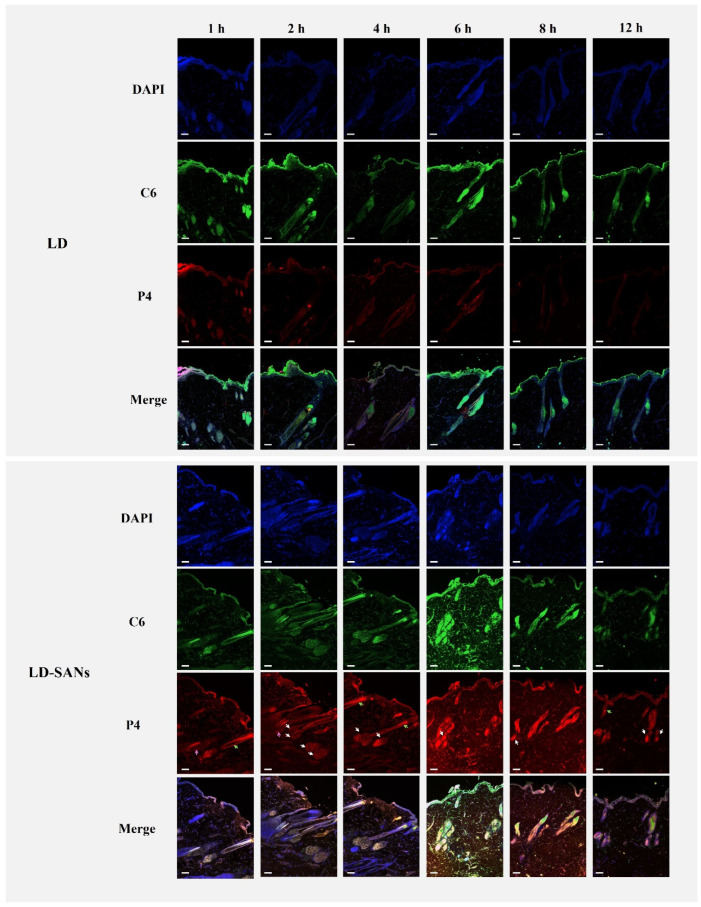
CLSM images of SD rat skin treated with LD and LD-SANs at different time points. Fluorescence images of C6 and P4 in vertical skin sections. Green, white, and purple arrows, respectively, capture typical hair follicles, sebaceous glands, and sweat glands. Scale bar = 50 μm.

Vertical sections of the skin showed that the C6-labeled LD and LD-SANs were first detected in hair follicles and the SC at 1 h. The diffusion of C6 fluorescence out of the follicles into the adjacent tissues started at 2 h, with the intensity of LD-SANs being much higher in comparison with LD, which signifies a higher nanoparticle abundance (Figure 5). The C6 signal of LD-SANs reached its maximum at 6 h and was distributed evenly in the dermis and then decreased, which was probably caused by clearance of the compounds. In the case of the P4 signal, the LD nanoparticles were mainly confined to hair follicles during the experiment and were observed to be weakly fluorescent, indicating that the bigger particles do not penetrate well. Conversely, LD-SANs-P4 had a strong fluorescence with deep penetration through follicles, sebaceous glands, and SC after 1 h. It was found to achieve peak distribution between layers of the skin and skin appendages at 6 h, and it showed better skin penetration, faster delivery, and higher retention of the drug compared to LD, which is consistent with ex vivo results.

#### 3.7.2. Analysis of Horizontal Skin Sections

**Figure 6:**
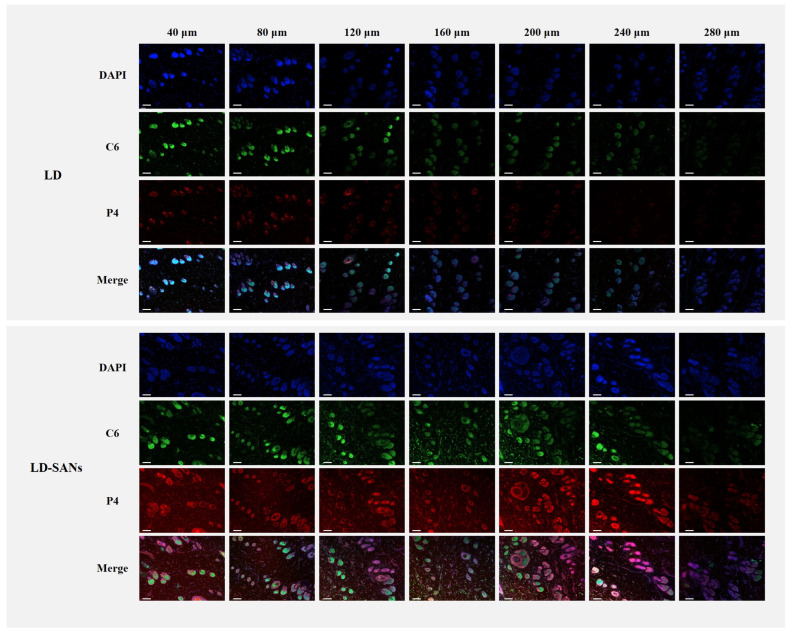
CLSM images of abdominal skin from SD rats at 8 h after in vivo administration of LD and LD-SANs. Fluorescence probe images of C6 and P4 in horizontal sections of rat skin. The figures show CLSM images of P4 and C6 in rat skin from the epidermis to the dermis at a distance of 280 μm. Scale bar = 50 μm.

In order to assess nanoparticle penetration further, horizontal skin sections were examined 8 h after administration. In agreement with the results of vertical sections, C6 (green) distribution was associated with the localization of nanoparticles (red) [30]. Fluorescence was largely localized to hair follicles in LD, and there was low signal intensity around follicles at a depth of 40 μm, which may be caused by the increased lipid fluidity of SC caused by sustained contact [31,32]. Fluorescence in deeper layers (≥80 μm) remained faint and localized to hair follicles, indicating limited penetration that is likely attributable to the low number and large size of the LD nanoparticles. In contrast, LD-SANs exhibited uniform, intense fluorescence throughout the skin layers, suggesting additional penetration pathways such as sebaceous glands, sweat glands, and adipose tissue (Figure 6). The strongest signal was observed in hair follicles, with high-intensity P4 and C6 fluorescence consistently detected from 40 μm to 280 μm, confirming the superior trans-follicular permeability of LD-SANs compared to LD.

#### 3.7.3. Analysis of Immunofluorescence Staining of CD207

The relevant literature has demonstrated that SANs are the ideal delivery system to follicles, as they can enter deeper into hair follicles compared to molecules in solution and reach perifollicular APCs without destroying the SC barrier [33,34]. Such APCs include specialized dendritic cells (DCs) that are found in the epidermal layer of the skin. Langerhans cells (LCs) are important elements of the skin immune system; these cells are involved in antigen presentation and immune regulation and cover 1–3% of epidermal cells. Nevertheless, they occupy almost 20–25 percent of the epidermal surface area because of their reticular structure, which allows them to capture any antigens that they encounter [35,36]. Thus, the lack of an SC barrier at the inferior orifice of hair follicles and capturing by perifollicular APCs could both contribute to the transdermal penetration of nanoparticles that permeate into the viable epidermis and settle in hair follicles [37]. We therefore labeled the APCs using IF to assess the potential interaction with the SANs.

**Figure 7:**
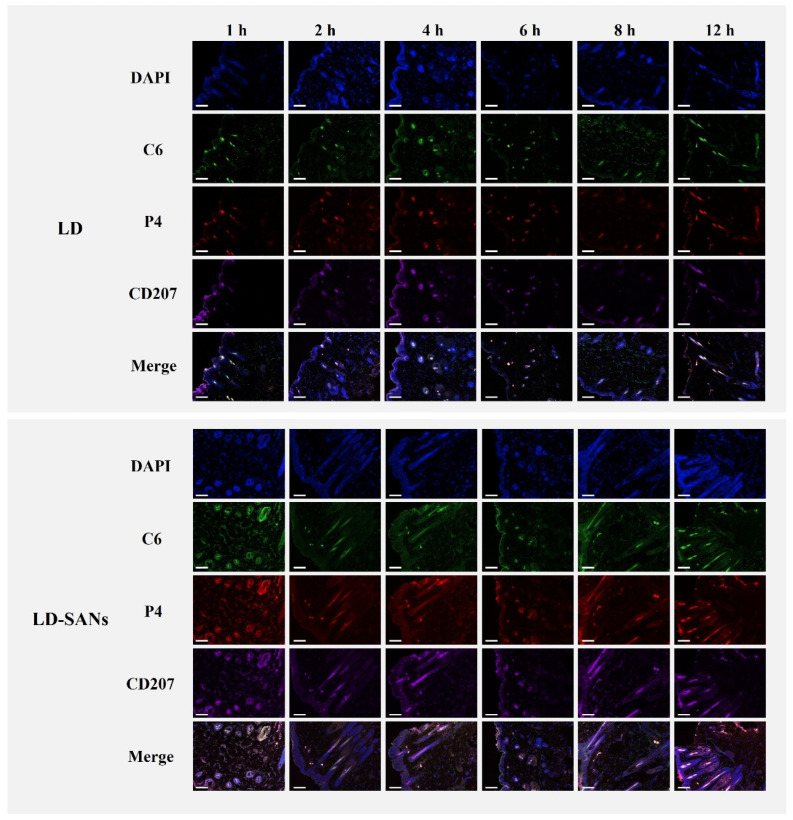
CLSM images of vertical cross-sections showing in vivo transdermal penetration of LD and LD-SANs in SD rats at different time points. The slides were stained with IF. Scale bar = 50 μm.

CLSM of vertical skin sections that received SANs found that the red fluorescence (SANs) was concentrated mainly in hair follicles, which showed that the transfollicular penetration pathway was the major route of delivery instead of transepidermal delivery (Figure 7). The release of C6 (green) was also observed to be diffused in perifollicular areas. The LD-SANs showed better skin penetration, based on the intense magenta/white signal overlap (red—SANs, purple—LCs, green—C6, and blue—nuclei) in large follicular and perifollicular areas, and it was much more intense compared to LD. Conversely, the LD nanoparticles were mainly located in the follicular lumen, and the red fluorescence was encircled by the purple LC signal, and there was little co-localization in the perifollicular area. This minor translocation can be explained by the fact that the LD nanoparticles were larger and accumulated less, and thus hindered LC capture. However, a small fraction reached the perifollicular area, but it was insignificant. All these findings indicate that LD-SANs are better captured by LCs in the epidermis and follicular areas and show excellent follicular translocation compared with LD.

### 3.8. Pharmacodynamic Experiment on Skin

**Figure 8:**
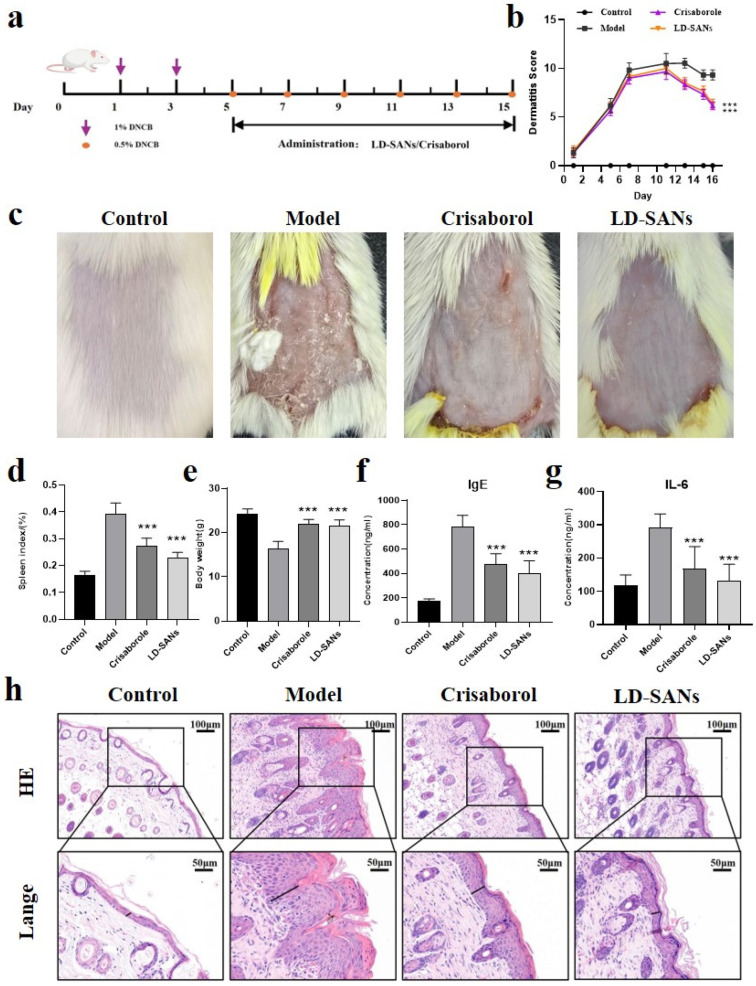
Chronic dermatitis/eczema mouse model. (**a**) Flow chart of dermatitis mouse model establishment. (**b**) Comparison of EASI scores of skin lesions in each group of mice. (**c**) Skin lesions of AD model mice in each group. (**d**) Spleen index of mice after four treatments. (**e**) Weight of body after four treatments. (**f**,**g**) Cytokine IgE and IL-6 expression in the chronic dermatitis mouse model. (**h**) H&E-stained images of mouse skin tissues from different treatment groups. The row labelled “Lange” denotes a local magnified view of the region of interest. Scale bar: 50 μm/100 μm. ANOVA followed by Dunnett’s multiple comparison test was performed to compare each condition against the treatment condition with the model (*** *p* < 0.001).

Figure 8b summarizes the scores of skin lesions. At baseline, the scores were zero, which increased to the maximum on day 11, showing that the models were induced successfully, and declined on day 16 in both the LD-SANs and positive control groups. There was apparent erythema, edema, and scabbing in the model group (Figure 8c), as well as reduced body weight and increased spleen index (Figure 8d,e). Conversely, the positive control and the LD-SANs groups were characterized by resolved or abated skin lesions, and better body weight and spleen index in line with the trends of the EASI score. ELISA findings showed that serum IgE and IL-1β were highly increased in the model group, and they were significantly decreased by LD-SANs and positive control treatments (Figure 8f,g). H&E staining showed that the model group had hyperkeratosis, spongiosis, acanthosis, and dermal infiltration, whereas the LD-SANs group had less epidermal thickening and inflammatory cell infiltration (Figure 8h), which confirmed therapeutic improvement at the histological level.

**Figure 9:**
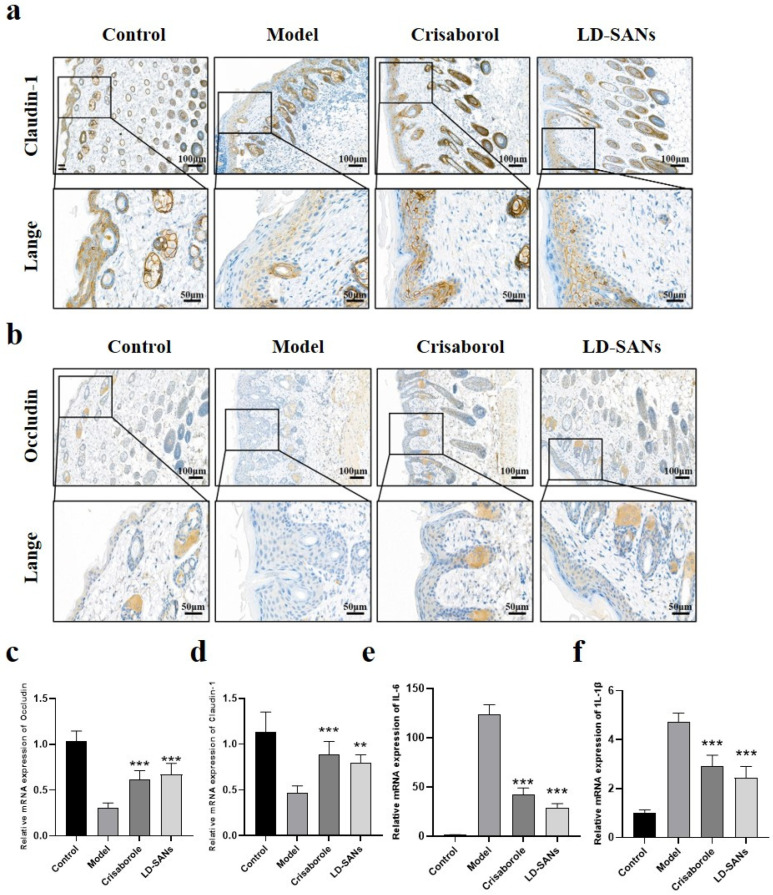
LD-SANs repair barrier proteins in AD mice. (**a**,**b**) IHC staining of Claudin-1 and Occludin. The rows labelled “Lange” denote a local magnified view of the region of interest. Scale bar: 50 μm/100 μm. (**c**–**f**) Transcription levels of the *Occludin*, *Claudin1*, *IL-6*, and *IL-1β* genes detected by RT-qPCR. All statistical data are expressed as means ± SD (*n* = 6). ANOVA followed by Dunnett’s multiple comparison test was performed to compare each condition against the treatment condition with the model (** *p* < 0.01, *** *p* < 0.001).

Tight junction (TJ) proteins, particularly Occludin and Claudin-1, play a vital role in ensuring skin barrier integrity through the regulation of permeability and inflammation [38]. The inhibition of these proteins may negatively affect TJ function and cause the expression of IL-1β in keratinocytes, thus enhancing epidermal inflammation [39]. The immunohistochemical examination showed that Occludin and Claudin-1 continuously and strongly stained along the membranes of the keratinocytes in the granular and upper spinous layers of mice in the blank group, which showed normal distribution of TJ (Figure 9a,b). Conversely, the model group showed fragmented and discontinuous staining, which is a sign of disrupted TJ integrity. The positive control and the LD-SANs groups demonstrated significantly enhanced staining with distinct intercellular junctions, indicating upregulated expression of Occludin and Claudin-1 and restored barrier localization. QPCR analysis demonstrated that LD-SANs significantly upregulated the expression of *Occludin* and *Claudin-1* (Figure 9c,d), which is consistent with immunohistochemical findings in tissue samples, suggesting the potential activation of skin barrier repair mechanisms. Previous studies indicate that certain AD therapeutics can act on keratinocytes to promote the transcription and translation of TJproteins, thereby enhancing skin barrier function [40,41,42]. Furthermore, LD-SANs may contribute to immune regulation by modulating T-cell subset functions, such as reducing the release of pro-inflammatory cytokines, including *IL-6* and *IL-1β*, through regulation of Th17 and other subsets (Figure 9e,f) [43,44]. It is also possible to reduce the recruitment of inflammatory cells, such as monocytes, by blocking the inflammatory signaling pathway [45]. In summary, LD-SANs likely alleviate AD symptoms by targeting core inflammatory pathways to restore skin barrier integrity and rebalance immune dysregulation.

## 4. Discussion

The clinical application of natural active components in TCM is limited by their poor solubility and low bioavailability [9,10]. Due to their diverse sources and unique structures, many active components of TCM can readily self-assemble into nano-formulations through various non-covalent interactions [9]. The formation of SANs not only modulates the absorption and distribution of active ingredients in TCM materials but also enhances the biological activity of these ingredients or their simple mixtures [46]. SANs have gained increasing popularity in the field of nanotechnology research due to their simplicity, environmental friendliness, and improved biodegradability and biocompatibility [47]. After the water decoction of licorice, intermolecular interactions occur among the chemical components of the TCM, leading to the formation of LD. However, LD exhibits large particle sizes and poor stability. In this study, the self-assembly process of traditional water decoctions was modified, and a systematic comparison was conducted between LD-SANs and LD. SEM images clearly demonstrated that the novel LD-SANs had a much smaller particle size than LD, with a uniform size and spherical structure. Additionally, the zeta potential of LD-SANs was higher than that of LD, indicating enhanced stability. The DL and EE of the active components in LD-SANs were both higher than those in LD, with the DL of small-molecule flavonoid components showing significant improvement. Meanwhile, spectral analysis and other analytical techniques were used to investigate the self-assembly process between LD and ethanol extraction. It was found that the electron cloud distribution of conjugated groups changed in both systems. The LD-SANs exhibited increased orderliness and improved thermal stability, which may be associated with the elevated crystallinity and a more regular crystal grain arrangement after the self-assembly process. In the LD-SANs system, hydrophobic flavonoids (e.g., LicA) drive core formation through π-π stacking, while Glycyrrhizin and related saponins align at the oil–water interface to lower interfacial energy. Polysaccharides and proteins from the aqueous extract further stabilize the nanoparticles via steric hindrance and hydrogen bonding. In conclusion, the novel LD-SANs effectively address the issues of large particle size, poor stability, and low DL of poorly soluble components in SANs derived from TCM.

Regarding the mechanism of the in vitro transdermal penetration experiment, our results showed that both LD-SANs and LD could penetrate effectively via the hair follicle pathway [48]. Licorice contains abundant saponin compounds, especially GA and its derivatives. These components are able to merge or displace SC lipids, break the close structure of lipid layers, and raise lipid fluidity. They can also interact with and aggregate with cell membrane cholesterol to create transient pores on the cell membrane, and introduce nanoparticles into cells by taking advantage of the permeabilizing and amphiphilic properties of saponins [49,50]. In addition, the primary active ingredients in the SANs, such as GA, glycyrrhetinic acid, liquiritigenin, and ammonium glycyrrhizinate, can also serve as drug delivery carriers in the self-assembly process [51,52]. The SANs form a closed film on the skin surface, increasing SC hydration. This leads to a decrease in the melting point of SC lipids and a reduction in the order of lipid tails, thereby promoting the loosening of lipid structures, significantly enhancing lipid fluidity, and facilitating the penetration of drug molecules through intercellular spaces [53,54]. Compared with LD, LD-SANs exhibited a higher cumulative penetration amount and faster penetration rate of the six active components. This may be attributed to the superior nanoparticle size of LD-SANs, along with their more stable structure and uniform distribution. The LD-SANs have higher DL and EE than LD, resulting in better solubility, and they can more easily pass through SC lipid gaps (approximately 20–50 nm) or hair follicle–sebaceous gland channels (pore diameter approximately 5–100 μm) to achieve “passive targeted penetration” [55,56]. The enhanced permeation of LD-SANs is attributed to a combination of passive diffusion via follicular pathways and saponin-mediated transient disruption of SC lipids. The sustained release of LicA from the hydrophobic core further contributes to prolonged skin retention, as supported by the higher Qs values in the LD-SANs group. Moreover, there was a significant difference in the retention amount of LicA between the two SANs. The novel preparation method significantly promoted the retention of LicA in the skin, which may be due to the fact that the hydrophobic component of LicA is largely encapsulated in the hydrophobic core of nanoparticles, avoiding degradation on the skin surface and enabling sustained release within the SC. At the same time, TCM components (such as GA and baicalin) may also directly inhibit drug-degrading enzymes in the skin (e.g., esterases), reducing the degradation of drugs by these enzymes, thereby increasing the retention amount and bioavailability of drugs in the skin [57,58].

Fluorescence analysis can be used to detect the location of penetrating nanoparticles in the epidermis and dermis of the skin. We found that the skin appendage pathway plays an important role in transdermal absorption [59], and the hair follicle pathway is the main route for SANs to penetrate deep into the skin [60,61]. Drug molecules can be released from SANs and diffuse into the surrounding dermal tissue where SANs are located. In the longitudinal sections of LD-SANs, we observed multiple times that intact skin accessory structures, such as hair follicles, sebaceous glands, and sweat glands, were filled with strong red fluorescence, which was relatively rare in the LD group. Further cross-sectional analysis showed that the fluorescence of LD significantly decreased at a depth of approximately 120 μm, while LD-SANs remained bright and uniformly distributed at 240 μm. In addition, the strong co-localization of LD-SANs and LCs in the living epidermis and hair follicle surrounding areas means that APCs are more likely to capture the nanoparticles. Hence, we can reasonably infer that LD-SANs have demonstrated excellent permeability and immune targeting potential in healthy rat models. In pathological conditions such as AD, the skin barrier is damaged and the SC structure is loose, accompanied by abnormal activation and enrichment of APCs. This microenvironment change is expected to further enhance the penetration and retention of SANs, thereby unlocking the enormous potential of LD-SANs in treating dermatitis.

In further pharmacodynamic research, LD-SANs show a good therapeutic effect on AD in mouse models, which can significantly improve the inflammatory and redness symptoms of AD in mice. This was manifested at the gene level by a decrease in *IL-1β*, along with an upregulation of the skin barrier proteins (*Occludin* and *Claudin-1*) in the administration groups. Genes encoding intercellular junction proteins (including Claudins and Occludins) belong to the genome responsible for the integrity and normal function of the epidermal barrier. These transmembrane and intracellular proteins form complexes connecting adjacent cells, called TJ. Located just below the SC, they prevent the diffusion of solutes through the intercellular spaces of simple and stratified epithelial cells, thereby regulating the selective permeability of the paracellular pathway [62,63]. In the epidermis, they are mainly located in the granular layer and are responsible for differentiation and keratinization. Impairment of these proteins leads to increased transepidermal water loss, dry skin, and infiltration and presentation of antigens by LCs [64,65]. Relevant studies have shown that AD patients exhibit decreased expression of TJproteins. The deficiency of Claudin-1 can mediate the impairment of TJ, which in turn leads to skin barrier dysfunction and immune dysregulation [66]. In this study, the results of IHC further verified that LD-SANs can exert pharmacodynamic effects by upregulating skin barrier proteins.

This study successfully developed LD-SANs, based on the co-assembly of aqueous and ethanol extracts of licorice, and conducted an in-depth investigation into their mechanism for enhancing skin permeability. The results showed that LD-SANs can effectively regulate the microstructure of the skin and increase the lipid fluidity in the SC, thereby promoting intercellular permeation. Through fluorescence tracing observation, we determined that hair follicles are the main pathway. Due to the ideal size and stability of LD-SANs, they are more easily captured by APCs around the hair follicles (such as LCs), showing a promising prospect for topical treatment. The superior therapeutic efficacy of LD-SANs in an AD mouse model was ultimately attributed to this enhanced skin penetration and its dual action of alleviating inflammation (e.g., downregulating IL-1β) and restoring skin barrier integrity (e.g., upregulating Occludin and Claudin-1).

## 5. Conclusions

This study holds dual significance. From a practical application perspective, transforming traditional herbal decoctions into high-performance nanomedicines directly addresses long-standing challenges such as instability and poor permeability. From a theoretical standpoint, guiding the self-assembly of natural products provides a new perspective for drug development, and the LD-SANs platform has great potential for future research and development. The precise molecular interactions and driving forces behind the synergistic assembly of water-soluble and alcohol-soluble components merit further in-depth study. Expanding the production scale and conducting long-term safety assessments will be key steps in applying this promising nano-platform to clinical translation. In summary, LD-SANs not only perform well in the long-term management of inflammatory skin diseases but also achieve the modernization of traditional drugs through nanotechnology.

## Figures and Tables

**Table 1:** EE of LD and LD-SANs ($\bar{x}\;\pm$ SD, *n* = 3).

Sample	Entrapment Efficiency/%					
	AG	GL	AGly	IG	GA	LicA
LD	56.34 ± 0.6515	54.90 ± 0.8364	43.74 ± 0.2403	55.69 ± 0.4979	55.39 ± 0.5130	43.21 ± 0.3631
LD-SANs	90.25 ± 0.2668	86.67 ± 0.1767	75.91 ± 0.4148	91.79 ± 0.8512	91.59 ± 0.6047	55.48 ± 0.2163

**Table 2:** DL of LD and LD-SANs ($\bar{x}\;\pm$ SD, *n* = 3).

Sample	Drug Loading Capacity/%					
	AG	GL	AGly	IG	GA	LicA
LD	75.08 ± 0.0039	61.92 ± 0.0050	20.29 ± 0.0007	35.93 ± 0.0026	56.71 ± 0.0039	7.09 ± 0.0004
LD-SANs	82.38 ± 0.0006	73.92 ± 0.0029	27.10 ± 0.0008	60.86 ± 0.0037	62.63 ± 0.0032	48.68 ± 0.0004

**Table 3:** In vitro transdermal parameters of LD and LD-SANs ($\bar{x}\;\pm$ SD, *n* = 3).

Components	Sample	*Qs*/(μg·cm^−2^)	*Js*/(μg∙cm^−2^∙h^−1^)	*Kp*/(×10^−3^ cm∙h^−1^)	Fitting Equation
AG	LD	21.84 ± 2.75	7.02 ± 1.67	0.90 ± 0.22	Q = 6.01198t + 39.67829
	LD-SANs	13.91 ± 4.68	10.75 ± 4.66	1.26 ± 0.55	Q = 9.47577t + 43.86897
GL	LD	4.26 ± 0.32	1.67 ± 0.23	1.51 ± 0.21	Q = 1.26288t + 18.28388
	LD-SANs	3.10 ± 0.61	2.27 ± 0.55	1.73 ± 0.42	Q = 1.81882t + 19.43979
AGly	LD	0.67 ± 0.16	0.18 ± 0.02	2.25 ± 0.22	Q = 10.51265 × (1 − e^−0.03597×t^)
	LD-SANs	0.76 ± 0.12	0.24 ± 0.05	2.19 ± 0.47	Q = 1.55174 × t^0.51157^
IG	LD	0.52 ± 0.06	0.15 ± 0.01	2.94 ± 0.11	Q = 1.67082 × t^1/2^ − 3.81189
	LD-SANs	0.49 ± 0.06	0.19 ± 0.02	2.09 ± 0.21	Q = 10.19258 × (1 − e^−0.04221×t^)
GA	LD	25.74 ± 0.37	10.52 ± 0.52	2.31 ± 0.11	Q = 45.29596 × t^1/2^ + 188.73514
	LD-SANs	25.63 ± 1.30	11.61 ± 1.79	2.31 ± 0.36	Q = 6.06885t + 277.04712
LicA	LD	3.72 ± 0.49	0.92 ± 0.30	8.39 ± 2.75	Q = 5.49182 × t^1/2^ + 5.82452
	LD-SANs	30.63 ± 6.53	1.24 ± 0.40	1.69 ± 0.54	Q = 0.92691t + 16.39857

## Data Availability

The original contributions presented in this study are included in the article/Supplementary Material. Further inquiries can be directed to the corresponding authors.

## Supplementary Materials

The following supporting information can be downloaded at: https://www.mdpi.com/article/10.3390/pharmaceutics18060661/s1, Figure S1. Regression equations of active components in LD-SANs; Figure S2. Molecular structure of P4; Figure S3. Fluorescence Stability. (a) Fluorescence P4 preservation rate of LD in different solution systems. (b) Fluorescence C6 preservation rate of LD in different solution systems. (c) Fluorescence P4 preservation rate of LD-SANs in different solution systems. (d) Fluorescence C6 preservation rate of LD-SANs in different solution systems. All statistical data are expressed as means ± SD (*n* = 3); Table S1. Determination of Particle Size for Different SANs ($\bar{x}$ ± SD, *n* = 3); Table S2. Determination of Particle Size Stability of SANs ($\bar{x}$ ± SD, *n* = 3); Table S3. Determination of Active Component Content in Each LD Group ($\bar{x}$ ± SD, *n* = 3); Table S4. Determination of Active Component Content in Each LD-SANs Group ($\bar{x}$ ± SD, *n* = 3); Table S5. Simulation and Fitting of Transdermal Cumulative Permeation Quantity Curve ($\bar{x}$ ± SD, *n* = 3); Table S6. Precision evaluation of LD-SANs; Table S7. Repeatability test of LD-SANs; Table S8. Stability study of LD-SANs; Table S9. The recoveries results of active components in LD-SANs (*n* = 3).
