# Supplementary material for: Self-Assembled Nanoparticles of Licorice Extract Enhance Skin Penetration and Regulate Barrier Proteins via a Dual-Channel Pathway
Source: Pharmaceutics. 2026 May 27;18(6):661. doi: 10.3390/pharmaceutics18060661 (PMC13304804; doi:10.3390/pharmaceutics18060661)
Supplement: Supplementary file 1 [file pharmaceutics-18-00661-s001.zip › pharmaceutics-4299391-supplementary.pdf]

Supplementary Materials

Figure S1. Regression equations of active components in LD-SANs

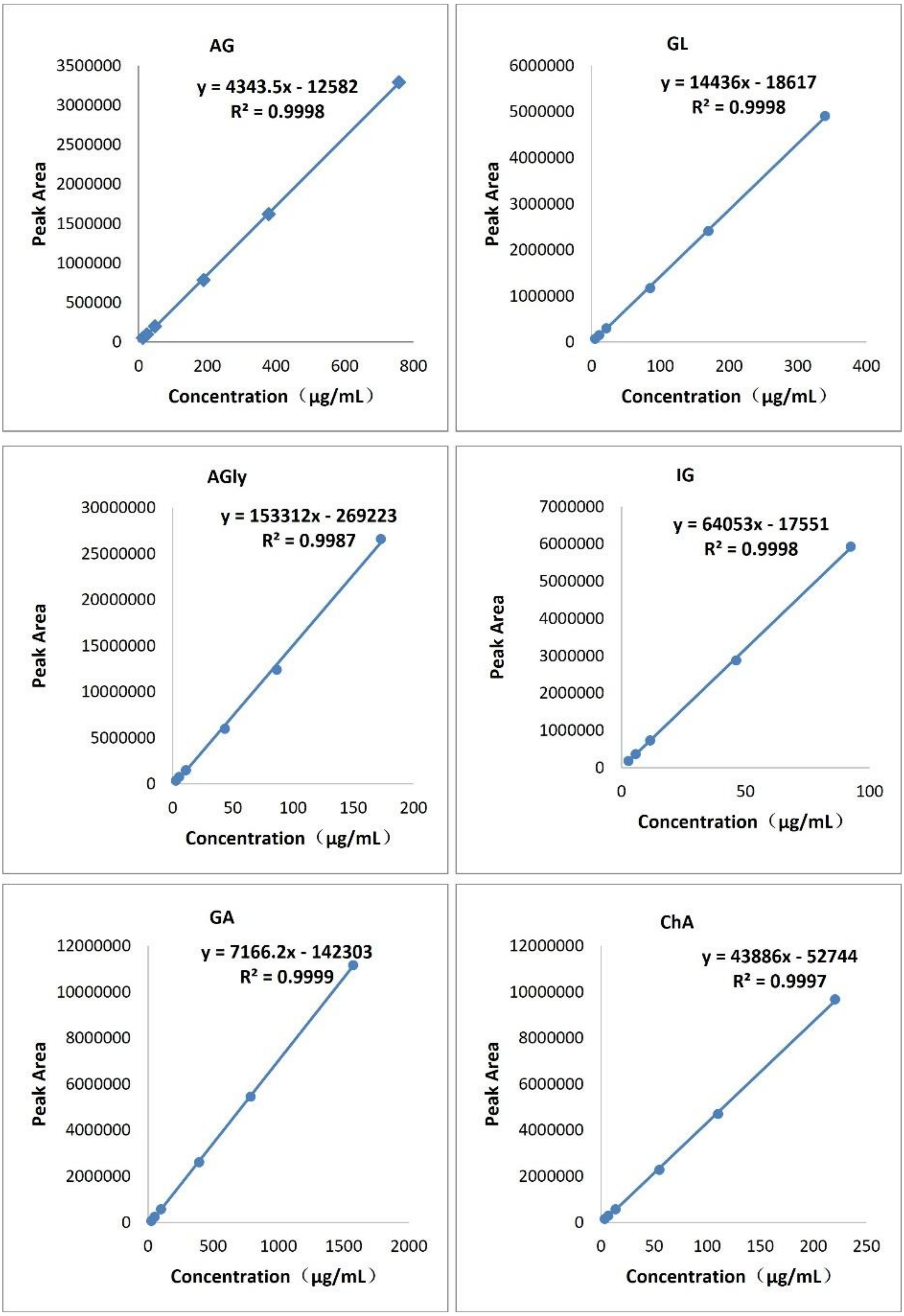

Figure S2. Molecular structure of P4

(2Z)-7-methoxy-2-[(7-methoxy-4,5-dihydro-1H-benzo[g]indol-2-yl)methylene]-4,5-dihydrobenzo[g]indole boron difluoride.

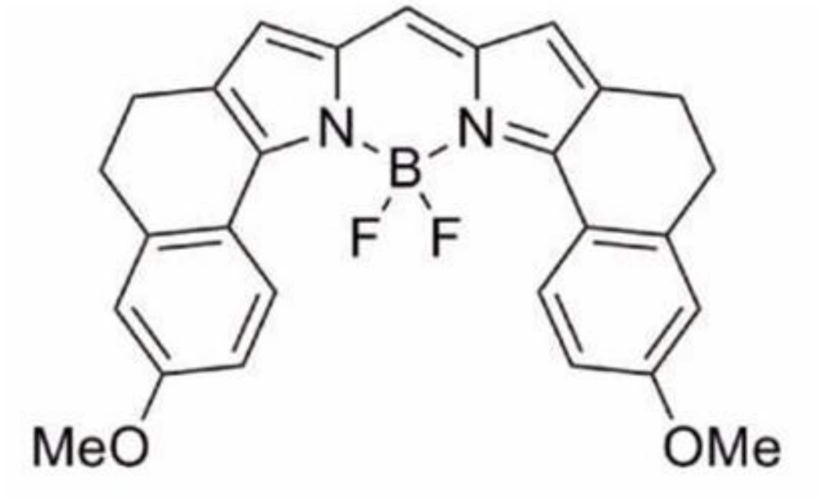

Figure S3. Fluorescence Stability. (a) Fluorescence P4 preservation rate of LD in different solution systems. (b) Fluorescence C6 preservation rate of LD in different solution systems. (c) Fluorescence P4 preservation rate of LD-SANs in different solution systems. (d) Fluorescence C6 preservation rate of LD-SANs in different solution systems. All statistical data are expressed as means ± SD (n = 3).

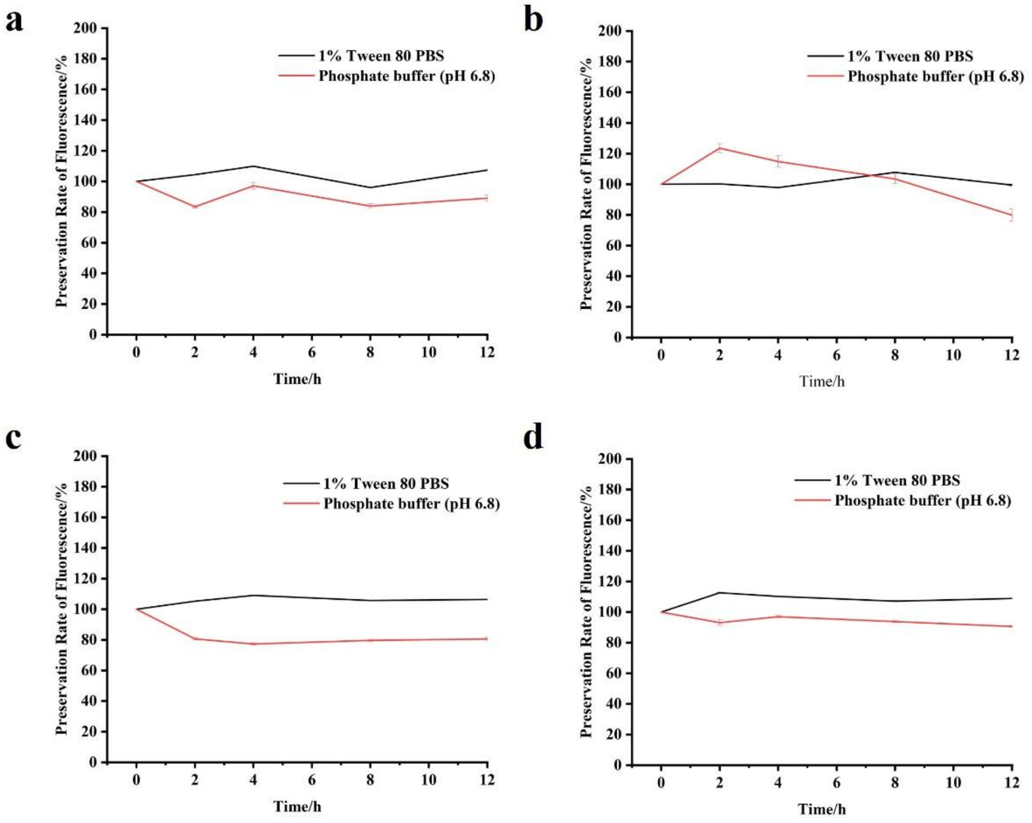

Table S1. Determination of Particle Size for Different SANs ($\bar{x} \pm$ SD, n=3).

Parameter	LD	LD-SANs
Size（nm）	544.00±20.42	129.60±5.96
PDI	0.86±0.09	0.20±0.10
ζ（mV）	33.18±0.67	68.85±0.42

Table S2. Determination of Particle Size Stability of SANs ($\bar{x} \pm$ SD, n=3).

Time（d）	LD		LD-SANs	
	Size (nm)	PDI	Size (nm)	PDI
1	544.00±20.42	0.86±0.09	129.60±5.96	0.20±0.10
2	567.67±20.55	0.70±0.08	144.07±3.94	0.13±0.09
3	557.00±14.42	0.82±0.16	167.40±7.83	0.25±0.08
4	759.33±38.55	0.98±0.23	181.53±3.87	0.18±0.07
5	939.67±19.55	0.84±0.20	151.67±7.78	0.14±0.09
6	973.33±1.53	0.92±0.22	194.13±5.04	0.16±0.03
7	1196.00±158.77	0.78±0.15	193.57±14.34	0.20±0.12

Table S3. Determination of Active Component Content in Each LD Group ($\bar{x} \pm$ SD, n=3).

Components / (mg·mL^{-1})	True Solution fraction	Nanoparticle fraction	Precipitate fraction	Whole Solution
AG	1.079±0.0245	4.376±0.0302	0.234±0.0028	7.767±0.0406
GL	0.184±0.0049	0.603±0.0043	0.038±0.0002	1.099±0.0089
AGly	0.006±0.0000	0.037±0.0002	0.003±0.0000	0.083±0.0003
IG	0.002±0.0000	0.03±0.0002	0.002±0.0000	0.054±0.0004
GA	0.225±0.0014	2.518±0.0170	0.145±0.0048	4.546±0.0314
ChA	0.000±0.0000	0.046±0.0001	0.016±0.0001	0.106±0.0006

Table S4. Determination of Active Component Content in Each LD-SANs Group ($\bar{x} \pm$ SD, n=3).

Components / (mg·mL^{-1})	True Solution fraction	Nanoparticle fraction	Precipitate fraction	Whole Solution
AG	0.734±0.0053	7.690±0.0183	0.201±0.0086	8.521±0.0061
GL	0.139±0.0009	1.137±0.0023	0.038±0.0015	1.312±0.0051
AGly	0.005±0.0000	0.085±0.0003	0.004±0.0001	0.111±0.0003
IG	0.002±0.0000	0.084±0.0004	0.003±0.0001	0.091±0.0005

GA	0.114±0.0008	4.598±0.0137	0.133±0.0071	5.020±0.0258
ChA	0.003±0.0000	0.405±0.0013	0.134±0.0050	0.730±0.0006

Table S5. Simulation and Fitting of Transdermal Cumulative Permeation Quantity Curve ($\bar{x} \pm$ SD, n=3).

Components	Sample	Zero-Order Equation	R^2	First-Order Equation	R^2	Higuchi Equation	R^2	Ritger Peppas Equation	R^2
AG	LD	Q=6.01198t+39.67829	0.99427	$Q=614.19237*(1-e^{-0.01558*t})$	0.95678	$Q=53.54812*t^{1/2}-65.61121$	0.94394	$Q=16.5626*t^{0.76698}$	0.97795
	LD-SANs	Q=9.47577t+43.86897	0.99142	$Q=1255.40275*(1-e^{-0.01046*t})$	0.97011	$Q=55.18116*t^{1/2}+148.82988$	0.98172	$Q=19.87707*t^{0.82948}$	0.98166
GL	LD	Q=1.26288t+18.28388	0.99702	$Q=89.73513*(1-e^{-0.03708*t})$	0.89145	$Q=11.31897*t^{1/2}-4.13872$	0.96059	$Q=8.15251*t^{0.57636}$	0.96944
	LD-SANs	Q=1.81882t+19.43979	0.99344	$Q=138.96796*(1-e^{-0.02782*t})$	0.93042	$Q=16.30979*t^{1/2}-12.88817$	0.95822	Q=8.27414*t0.65248	0.9753
AGly	LD	Q=0.17624t+1.16477	0.78993	$Q=10.51265*(1-e^{-0.03597*t})$	0.90516	$Q=1.6984*t^{1/2}-2.47804$	0.90459	$Q=0.65093*t^{0.68326}$	0.85755
	LD-SANs	Q=0.17806t+3.21323	0.9629	$Q=11.68038*(1-e^{-0.05217*t})$	0.93054	$Q=1.63998*t^{1/2}-0.13877$	0.9888	$Q=1.55174*t^{0.51157}$	0.98888
IG	LD	Q=0.17699t-0.30414	0.75258	Q=16.82588*(1-e-	0.76519	$Q=1.67082*t^{1/2}-3.81189$	0.82116	$Q=0.20927*t^{0.93568}$	0.75116

				0.01228*t)					
	LD-SANs	Q=0.17223t+1.61202	0.79898	$Q=10.19258*(1-e^{-0.04221*t})$	0.92844	$Q=1.65989*t^{1/2}-1.94853$	0.91493	$Q=0.82263*t^{0.62979}$	0.88063
GA	LD	Q=4.96326t+280.36563	0.97872	$Q=438.84975*(1-e^{-0.16441*t})$	0.4663	$Q=45.29596*t^{1/2}+188.73514$	0.98369	$Q=189.41021*t^{0.24722}$	0.9537
	LD-SANs	Q=6.06885t+277.04712	0.98526	-		$Q=55.18116*t^{1/2}+165.89068$	0.98172	$Q=175.81585*t^{0.28959}$	0.95466
ChA	LD	Q=0.60049t+16.96071	0.984	$Q=42.22972*(1-e^{-0.07397*t})$	0.8205	$Q=5.49182*t^{1/2}+5.82452$	0.99388	$Q=9.1911*t^{0.40202}$	0.9864
	LD-SANs	Q=0.92691t+16.39857	0.99376	$Q=63.05405*(1-e^{-0.04707*t})$	0.90416	$Q=8.40559*t^{1/2}-0.48184$	0.98406	$Q=7.74776*t^{0.52134}$	0.98543

Table S6. Precision evaluation of LD-SANs.

Number	AG	GL	AGly	IG	GA	ChA
1	2600864	3834550	19697573	9294099	8427057	7447246
2	2586224	3812265	19565602	9252577	8372148	7407759
3	2582087	3811478	19522435	9222569	8360015	7382194
4	2622121	3860710	19873656	9372655	8508822	7468599
5	2531738	3734333	19095449	9041594	8199211	7232668

6	2518158	3710934	18994565	8996769	8156102	7186407
Mean	2573532	3794045	19458213	9196711	8337226	7354146
SD	40386	58641	344125	147152	134987	116894
RSD/%	1.57	1.55	1.77	1.60	1.62	1.59

Table S7. Repeatability test of LD-SANs.

Number	AG	GL	AGly	IG	GA	ChA
1	1979020	980847	663399	310413	1871824	1785370
2	1963992	972078	657868	308844	1863155	1772823
3	1954730	971518	654861	306485	1847329	1761582
4	1947126	967946	651884	305867	1853946	1754870
5	1933300	957766	647674	303929	1827875	1742786
6	1978010	983859	661192	311122	1867631	1776718
Mean	1959363	972336	656146	307777	1855293	1765692
SD	17922	9354	5876	2807	16151	15610
RSD/%	0.91	0.96	0.90	0.91	0.87	0.88

Table S8. Stability study of LD-SANs.

Number	AG	GL	AGly	IG	GA	ChA
0h	1975391	976697	661030	310274	1863459	1780514
2h	1964826	975408	659052	309173	1867350	1774496
4h	1935854	964675	649017	303961	1838605	1746162
8h	1936066	967367	647820	304769	1819067	1762327
12h	1968012	976891	659489	309698	1852627	1768767
24h	1979238	981761	660765	310428	1856437	1781270
Mean	1959898	973800	656196	308051	1849591	1768923
SD	19237	6458	6082	2900	17979	13262
RSD/%	0.98	0.66	0.93	0.94	0.97	0.75

Table S9. The recoveries results of active components in LD-SANs (n=3）.

Components	Sample Content /ug	Spiked Amount /ug	Measured Amount /ug	Recovery Rate /%	Average Recovery Rate /%	RSD/%
AG	376.40	420.34	396.25	98.99	99.62	0.93

	362.32	420.34	389.60	99.18		
	388.69	420.34	405.96	100.69		
	369.80	358.67	358.76	96.95		
	369.52	358.67	357.70	96.44	97.36	1.22
	360.71	358.67	357.37	98.71		
	378.78	263.33	320.03	99.22		
	375.66	263.33	318.87	99.53	98.53	1.49
	379.49	263.33	317.25	96.84		
	49.87	67.65	59.57	102.40		
	51.15	67.65	61.51	106.25	104.48	1.86
	52.22	67.65	61.55	104.78		
	52.00	50.93	50.53	96.32		
GL	48.75	50.93	49.27	97.75	96.48	1.23
	51.11	50.93	49.84	95.38		
	57.66	39.74	49.55	104.29		
	57.29	39.74	49.50	104.93	104.71	0.35
	57.93	39.74	49.81	104.90		

	5.10	7.83	6.36	97.33		
	5.08	7.83	6.27	95.10	95.68	1.52
	5.03	7.83	6.22	94.59		
	5.10	7.04	5.97	97.28		
AGly	5.18	7.04	5.94	95.01	95.66	1.47
	5.15	7.04	5.91	94.69		
	5.17	5.76	5.37	96.67		
	5.13	5.76	5.30	95.03	95.53	1.04
	5.14	5.76	5.30	94.89		
	3.71	5.65	4.54	95.04		
	3.65	5.65	4.54	96.14	95.29	0.79
	3.71	5.65	4.53	94.70		
	3.70	4.57	4.04	95.82		
IG	3.74	4.57	4.09	97.05	96.19	0.78
	3.79	4.57	4.08	95.71		
	3.81	3.58	3.60	94.50	95.42	0.99
	3.80	3.58	3.63	96.38		

	3.89	3.58	3.65	95.37		
	216.76	265.57	241.28	100.08		
	213.63	265.57	240.19	100.44	99.97	0.53
	215.87	265.57	239.92	99.40		
	215.46	213.67	211.48	97.11		
GA	224.05	213.67	215.94	97.27	97.16	0.10
	221.32	213.67	214.38	97.09		
	222.77	135.46	181.68	103.78		
	228.30	135.46	184.55	103.95	102.80	1.79
	233.21	135.46	184.79	100.67		
	31.64	40.41	35.60	97.91		
	31.80	40.41	35.20	95.55	96.26	1.49
	32.17	40.41	35.34	95.32		
ChA	32.06	33.42	32.15	96.50		
	33.35	33.42	33.14	98.53	98.04	1.40
	33.31	33.42	33.22	99.11		
	33.71	31.75	32.67	99.63	99.49	0.15

	33.30	31.75	32.45	99.51
	34.87	31.75	33.21	99.34
